# Clinical study of intelligent tongue diagnosis and oral microbiome for classifying TCM syndromes in MASLD

**DOI:** 10.1186/s13020-025-01118-w

**Published:** 2025-05-29

**Authors:** Jialin Deng, Shixuan Dai, Shi Liu, Liping Tu, Ji Cui, Xiaojuan Hu, Xipeng Qiu, Hao Lu, Tao Jiang, Jiatuo Xu

**Affiliations:** 1 Department of College of Traditional Chinese Medicine, Shanghai University of Traditional Chinese Medicine, Shanghai, 201203 China; 2 School of Computer Science, Fudan University, 1200 Cailun Road, Pudong New Area, Shanghai, 201203 China; 3 Shuguang Hospital Affiliated to Shanghai University of Traditional Chinese Medicine, 1200 Cailun Road, Pudong New Area, Shanghai, 201203 China

**Keywords:** Metabolic dysfunction-associated steatotic liver disease (MASLD), TCM syndromes, Tongue diagnosis, Microbial metabolism, Machine learning

## Abstract

**Background:**

This study aimed to analyze the tongue image features and oral microbial markers in different TCM syndromes related to metabolic dysfunction-associated steatotic liver disease (MASLD).

**Methods:**

This study involved 34 healthy volunteers and 66 MASLD patients [36 with Dampness-Heat (DH) and 30 with Qi-Deficiency (QD) syndrome]. Oral microbiome analysis was conducted through 16S rRNA sequencing. Tongue image feature extraction used the Uncertainty Augmented Context Attention Network (UACANet), while syndrome classification was performed using five different machine learning methods based on tongue features and oral microbiota.

**Results:**

Significant differences in tongue color, coating, and oral microbiota were noted between DH band QD syndromes in MASLD patients. DH patients exhibited a red-crimson tongue color with a greasy coating and enriched *Streptococcus* and *Rothia* on the tongue. In contrast, QD patients displayed a pale tongue with higher abundances of *Neisseria*, *Fusobacterium*, *Porphyromonas* and *Haemophilus*. Combining tongue image characteristics with oral microbiota differentiated DH and QD syndromes with an AUC of 0.939 and an accuracy of 85%.

**Conclusion:**

This study suggests that tongue characteristics are related to microbial metabolism, and different MASLD syndromes possess distinct biomarkers, supporting syndrome classification.

## Instruction

Metabolic dysfunction-associated steatotic liver disease (MASLD). is characterized by a spectrum of diseases ranging from simple steatosis to advanced metabolic steatohepatitis with or without fibrosis. It may progress to cirrhosis and liver cancer, including an increased risk of other critical extrahepatic diseases [1]. As a common long-term liver disease, MASLD affects millions worldwide [2]. Liver steatosis and at least one of the three metabolic risk factors, including type 2 diabetes, obesity, or signs of metabolic dysregulation, are used to diagnose MASLD [1, 3].

Traditional Chinese medicine (TCM) is a holistic concept and a branch of conventional medicine in China. TCM regards “Gan Dan (liver disease)” as the TCM name for MASLD, and believes that its etiology and pathogenesis are improper diet, excessive comfort, emotional disorders, phlegm dampness constitution, and age and physical decline [4]. However, in the development process of MASLD, due to the different pathogenic factors and disease progression, it can be manifested as different TCM syndrome types. Although it belongs to the same disease, the differences between different syndromes also affect the outcome of the disease. Therefore, it is an effective clinical method to understand the differences between different syndromes and treat diseases based on syndrome differentiation.

Tongue diagnosis is an essential link in TCM diagnosis. TCM can identify the disease's nature and progression according to tongue image characteristics. TCM collecting data by visual observation cannot objectively or quantitatively summarise these features [5]. Modern tongue diagnosis technology is integrated with computers. At the same time, the depth of the study can be used to explain the tongue image comprehensively and get a description of the pathology [6]. In our previous study, we designed a complete set of image classification methods and applied tongue image analysis to the diagnosis of MASLD and diabetes mellitus and physical identification of the physical examination population [7–9], In addition, a large number of tongue images were evaluated by a deep learning model to establish optical information of tongue image features [10].

The tongue in the mouth at the same time as the first part of the digestive tract, oral microbiome with the normal oral correlation between ecological balance, and the correlation between the occurrence of systemic diseases and development [11]. In this pilot study of the project group, We have found that the tongue-coating *Streptococcus* and *Rothia*, intestinal *Blautia* and *Streptococcus* are potential biomarkers for MASLD [12]. However, to make an accurate diagnosis and treatment of MASLD, it is necessary to clarify the differences between different syndromes of MASLD.

TCM believes that there is an inseparable relationship between” tongue image-syndrome type -disease”. According to the changes in different syndrome types, even the same disease should be treated differently. However, rigorous statistical evidence for this view is lacking. There are many methods of syndrome differentiation in traditional Chinese medicine, among which syndrome differentiation based on deficiency and excess is one of the most important methods. MASLD is divided into deficiency syndrome and positive syndrome according to its etiology. The deficiency syndrome is mainly “qi-deficiency”, and the positive syndrome is mainly “dampness-heat”. This study takes “dampness-heat” syndrome and “qi-deficiency” syndrome, the two most common clinical MASLD syndromes, as the research objects, to explore the differences of tongue characteristics and oral-intestinal flora in different TCM syndromes, to provide a basis for achieving an accurate diagnosis of MASLD, contribute to achieving personalized medicine, and further strengthen the understanding of the scientific basis of TCM tongue diagnosis.

## Materials and methods

### Study design and subjects

Patients who attended the Department of Endocrinology and Physical Examination Center of Shuguang Hospital Affiliated with Shanghai University of Traditional Chinese Medicine from 2021.02 to 2021.12. According to the consensus opinion on the diagnosis and treatment of MASLD integrated traditional Chinese and Western medicine [4], the syndrome types of the patients were classified by senior Chinese medicine experts according to clinical manifestations, pulse, and tongue. After classification, the syndrome with a small number of people was excluded(Only 12 patients with Phlegm-Turbid syndrome and 6 patients with Phlegm-Stasis syndrome). Finally, the MASLD patients with Dampness-Heat(DH) syndrome and Qi-Deficiency(QD) syndrome were selected as the objects of follow-up experiments. The inclusion and exclusion criteria are shown in Table 1. Finally, 34 healthy volunteers and 66 patients with MASLD were included (36 with DH syndrome and 30 with QD syndrome). All the participants were requested to write the informed consent approved by the ethics committee of Shuguang Hospital, Affiliated with Shanghai University of TCM.

**Table 1:** Inclusion and exclusion criteria for subjects

Inclusion criteria	
Age 25–80 years MASLD Diagnostic Criteria [13]: Based on the evidence of hepatic fat deposition (histological, non-invasive biomarkers, or imaging), along with at least one of the following three conditions: (1) overweight or obesity; (2) type 2 diabetes; and (3) presentation of at least two metabolic dysfunction features	
Exclusion criteria	
History of heart diseases, such as heart failure, angina, and myocardial infarction History of malignant tumors or pulmonary diseases History of stroke or ischemic heart diseases History of taking probiotics or antibiotics within a month Oral diseases such as untreated oral abscesses or fungal infections Acute complications of type 2 diabetes Presence of problem with taking actigraphy for any reason	
TCM main syndrome evaluation scheme for MASLD [4]:	
Qi-Deficiency	(1) Thoracic rib fullness; (2) Depression and discomfort; (3) Fatigue; (4) Abdominal pain and diarrhea; (5) Tongue light red, thin white or white coating, tooth marks, and the fine pulse string
Dampness-Heat	(1) Yellowing of body and eyes; (2) Yellow urine color; (3) Stickiness in the mouth; (4) Dry mouth and bitter mouth; (5) The tongue is red in texture, the tongue coating is yellow and greasy, and the veins are smooth or moist
Phlegm-Turbid	(1) Obesity in body posture; (2) Discomfort or tightness in the right flank; (3) Overall fatigue and heaviness; (4) Sticky and uncomfortable stools; (5) The tongue is light in texture, with a white and greasy coating and smooth veins
Phlegm-Stasis	(1) Thoracic or dull pain in the ribs; (2) Subclavian lumps; (3) Dark complexion; (4) Body obesity; (5) The tongue is dark red with ecchymosis, and the body is plump with teeth marks on the edges. The coating is greasy, and the veins are smooth or astringent

### Data collection and analysis

#### Clinical data collection

The subject's names, ages, genders, Body Mass Index (BMI), and Waist-hip ratio (WHR)., Alanine Aminotransferase(ALT), Aspartate Aminotransferase (AST), Gamma-GlutamylTransferase (GGT), triglyceride(TG), High-density lipoprotein cholesterol(HDL-C), Low-density lipoprotein cholesterol(LDL-C), Total bilirubin(TBIL), Direct bilirubin(DBIL), Indirect bilirubin(IBIL), Fasting blood glucose(FBG),2 h postprandial blood glucose(2 hPG), Glycosylated hemoglobin (HbA1 C)were collected from the subjects.

#### Tongue diagnosis

**Fig. 1:**
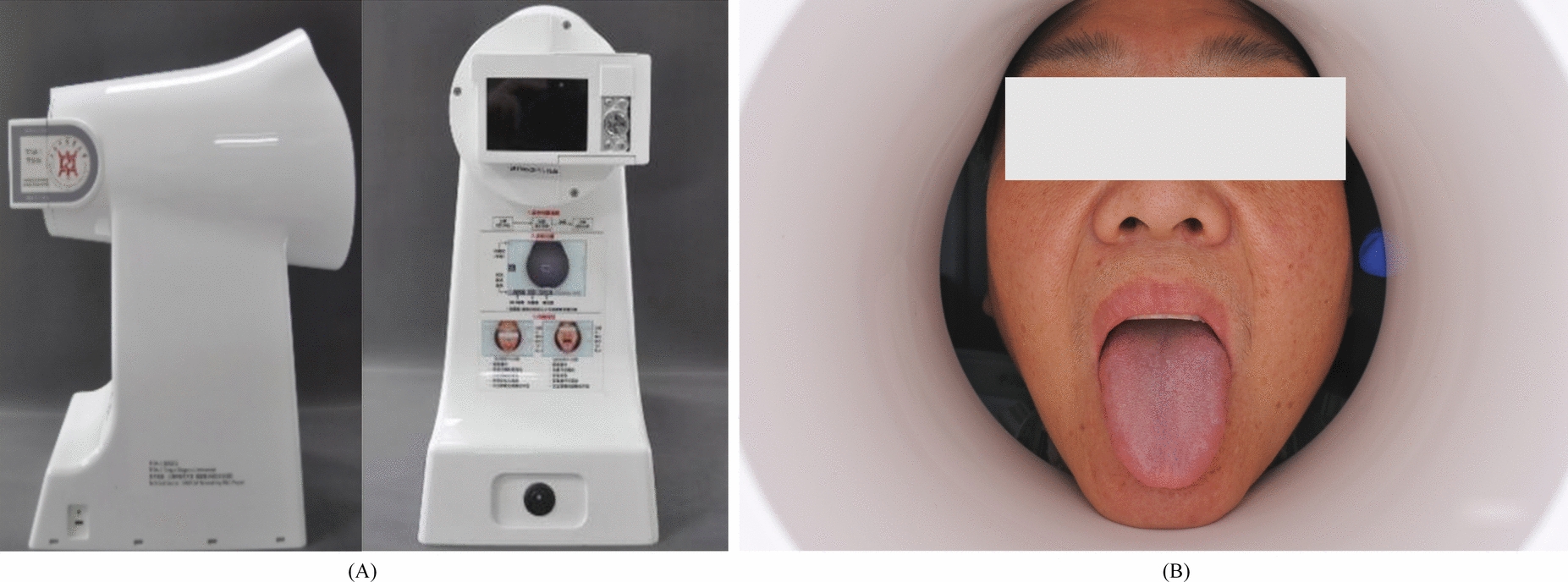
Display of TFDA-1 tongue diagnostic instrument. **A** is the device diagram of TFDA-1, **B** is the shooting interface picture

The collection equipment used was the Tongue Diagnostic Instrument (TFDA-1) developed by the Intelligent Diagnostic Laboratory of the Shanghai University of Traditional Chinese Medicine. TFDA-1 tongue diagnostic instrument is shown in Fig. 1. Main technical parameters: Manual Mode; shutter speed: 1/125; aperture value: F6.3; ISO sensitivity: 200; color temperature: 4500 K–6000 K; illumination 4800 ± 10% (unit: lx). The procedure for tongue image acquisition and quality control is as follows: ① Environmental Control and Equipment Disinfection: The image acquisition is conducted under controlled room temperature and natural lighting conditions. The imaging parameters are set accordingly, and the device is disinfected with alcohol before use; ② Standardized Subject Preparation: Subjects are instructed to refrain from eating, smoking, or consuming colored beverages for at least 15 min before image acquisition. Additionally, they are required to rinse their mouths with warm water to remove any food residues and excess saliva; ③ Image Acquisition: Under the guidance of the researcher, the subject places their mandible on the chin rest of the tongue diagnosis device, opens their mouth, relaxes the tongue naturally, flattens it with the tongue tip pointing downward, and quickly aligns the center of the tongue surface with the device’s camera to complete the acquisition process. The detailed acquisition procedure is referenced in previous work [14]; ④ Image Quality Evaluation: A ResNet-based classification model, developed in previous studies, is employed to assess and categorize the acquired tongue images [10]. Only images that meet the following criteria—intact tongue structure, proper positioning, absence of blurring, no light leakage, and no overexposure or underexposure—are included in the study. Images that fail to meet these requirements must be reacquired.

In this experiment, the tongue image feature extraction method is different from the previous work, such as Mask Region-based Convolutional Neural Network and generative adversarial network Tongue-GAN [10, 15]. We pay more attention to the accurate segmentation of the fuzzy part of the tongue base and use Uncertainty Augmented Context Attention Network (UACANet) to extract tongue image features.UACANet is an uncertainty-enhanced contextual attention network, which aims to improve the fusion and utilization efficiency of multi-scale features by introducing sensitive recognition of uncertain regions. Through the design of the dual-layer codec and prediction module, the recognition accuracy of the lesion area is greatly enhanced, and the segmentation of cysts and polyps has reached the industry-leading level [16, 17].

**Fig. 2:**
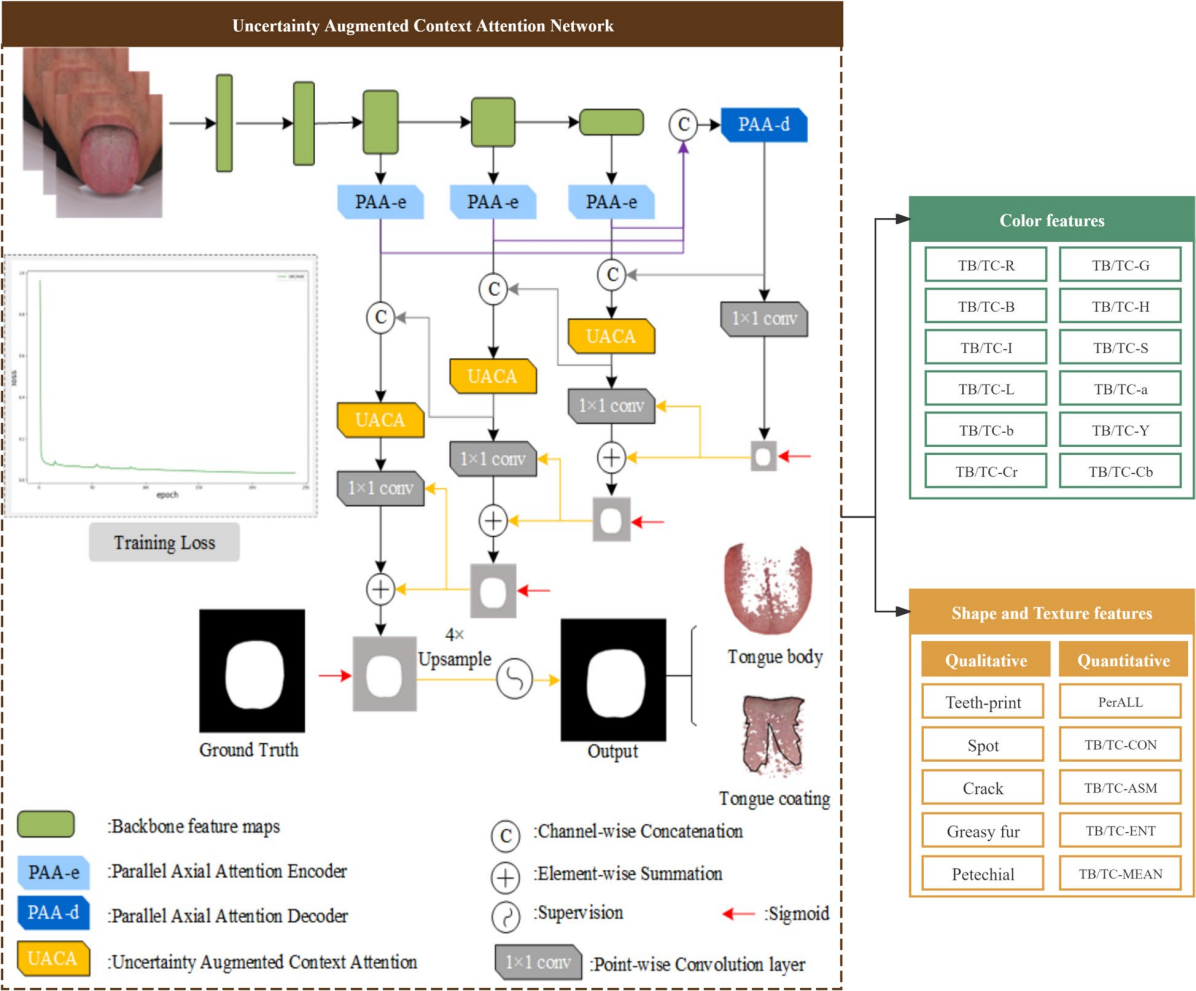
Intelligent tongue image analysis based on UACANet

UACANet not only improves the adaptability of the model to complex tissue structures but also strengthens the judgment of unclear edge regions. The figure below shows the entire process of tongue feature extraction using UACANet (Fig. 2). First, we randomly selected 1200 tongue surface images from the high-quality standardized clinical tongue image database constructed by our research group. Their original size was 5568*3712 DPI, and the label was used to label the fine-grained tongue body region and fine tongue classification features, of which 1000 tongue images were used as training data. Two hundred tongue images were used for the test data. Second, Adam is used as the backpropagation optimizer for the network parameters, where the learning rate is 1.0e-04, the Batch size is 8, and the epoch is 240. The results showed that the Uacanet-based tongue image segmentation model achieved 95.33% mIoU, mean ACC 0.985, and Dice coefficient 97.6% in the test dataset, indicating that the UACANet model could extract the fine-grained classification core features of images, and achieve high-precision tongue image segmentation and classification. Finally, the tongue and tongue coating regions were segmented by the “segmentation merge algorithm” and the “color threshold method”. After the segmentation, the color parameters of Lab color space, texture index, and tongue coating index were extracted according to the previous pattern recognition method. [12].

#### Oral microbial collection and analysis

Tongue coating samples were collected between 7:00 and 9:00 in the morning. All subjects provided samples on an empty stomach; if they had eaten, we asked them to rinse their mouths 2–3 times with normal saline for approximately 10 s each time. The subjects rinsed their mouths thoroughly and fasted for 1–2 h before collection. Collection method: The participants used a sterile throat swab to scrape the tongue coating from the middle part of the back of their tongue, rotating it with little force at least ten times to collect samples. After collection, we placed the swab head into a 2 ml sterile EP tube and quickly transported it on ice to an 80 °C refrigerator for storage until sequencing.

Bacterial DNA from the tongue dorsum and fecal samples were extracted using a swab genomic DNA extraction kit (CW2654, CwBiotech, Beijing, China) and an intestinal DNA extraction kit, respectively (TIANamp Stool DNA Kit, DP328, Tiangen Biotech, Beijing, China). The 16S rDNA full-length assembly sequencing technology (16S-FAST) was used to perform further classifications to reach a species level [18] by assaying DNA sequences encoding bacterial ribosomal 16S RNA, including 9 variable regions and 10 conserved regions. Qualitative and quantitative analyses as well as quality control were performed using 10 ng DNA. The splice and link libraries were then built. Next, data from electrophoresis and the measurements of Qubit concentrations were assembled for quality control before Illumina NovaSeq 6000 sequencing (Illumina, USA). The process was detailed in a previous study published by members of the research team [19].

We utilized a cloud platform (https://www.genescloud.cn/home) for the sequence analyses, including QIIME2 (2019.4), R language (v3.2.0), ggplot2 package, and Python. ASV-level alpha diversity indices and Shannon diversity index were calculated using the ASV table in QIIME2 and visualized as box plots. Kruskal–Wallis rank sum test and Dunn’s test were used as post-hoc tests to verify the significance of the difference. ASV-level ranked abundance curves were generated to compare the richness and evenness of ASVs among samples. Beta diversity analysis was performed to investigate the structural variation of microbial communities across models using UniFrac distance metrics and visualized via principal coordinate analysis (PCoA) hierarchical clustering. The significance of microbiota structure differentiation among groups was assessed by PERMANOVA (Permutational multivariate analysis of variance) using QIIME2. Linear discriminant analysis effect size (LEfSe) was used to detect the differentially abundant taxa among the groups using the default parameters. Random forest analysis was applied to discriminate the samples from different groups using QIIME2 with default settings. Nested stratified k-fold cross-validation was used for automated hyperparameter optimization and sample prediction. Co-occurrence network analysis was performed by SparCC analysis. The pseudo-count value in SparCC was set to 106. The cutoff of correlation coefficients was determined as 70 through random matrix theory-based methods as implemented in R package RMThreshold, and network visualization was constructed by Cytoscape (Cytoscape_v3.9.0). The R language was used to analyze the topological structure of the network. The key species were found according to the topological index, and the ZiPi diagram was used for visualization. PICRUSt2 (Phylogenetic Investigation of Communities by Reconstruction of Unobserved States) predicted the microbial function on MetaCyc (https://metacyc.org/).

### Machine learning methods

We used the logistic regression method with backward selection, called Logistic Regression, where L2 regularisation was used, tolerance was 1e-4, the inverse constraint factor C was 1.0, the iteration number max_iter was 100, and the lbfgs solver was used. We screened tongue image features and microbial data for syndrome classification prediction, eliminating unimportant variables and addressing multicollinearity [20]. Model fitness was assessed using maximum likelihood and the HL test. fivefold cross-validation was used to robustly assess model performance, dividing data into five subsets and averaging evaluation metrics such as the area under the curve (AUC), accuracy, sensitivity, and specificity [14, 21]. Aiming to capture non-linear relationships, we used Python 3.10.9 for machine learning, including support vector machine (SVM) and random forest. We used the sklearn (Version 1.3.1) library to calculate machine learning classification results.

### Statistical analysis

Data analysis was performed with SPSS v. 25.0 software (IBM Corp., Armonk, NY, USA). The normality of the variable distribution and homogeneity of variances were determined with the Shapiro–Wilk and Levene tests, respectively. In the case of normal distribution and homogeneity of variance, a t-test was used. Otherwise, a non-parametric test was performed. The comparison of categorical variables by Fisher’s exact test and Wilcoxon rank-sum test was applied to categorical variables and continuous variables. The associations between independent variables were analyzed via Spearman’s rank test, and the p-values were corrected via the Bonferroni correction for multiple comparisons.

## Result

### Characteristics of the patients

**Fig. 3:**
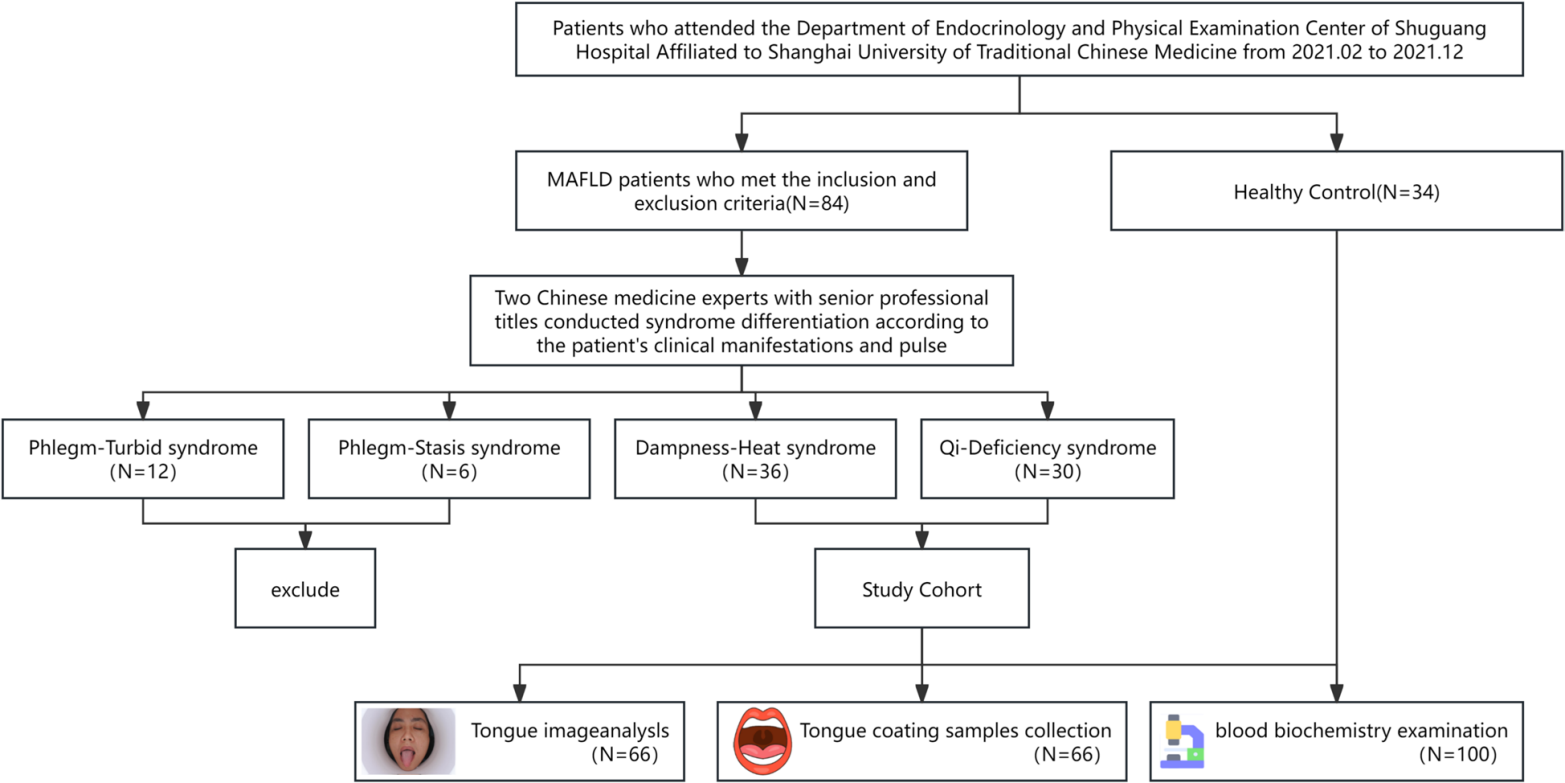
Program flowchart

The basic, biochemical, and microbiome composition-related information of 100 patients was collected. Of these, 34 were healthy controls and 66 were MASLD patients (36 with DH syndrome and 30 with QD syndrome). After screening 66 tongue imaging and tongue coating samples were collected (Fig. 3).

By exploring the demographic characteristics of the included population, it was found that there were significant differences in age, BMI, WHR, and blood pressure between the control group and the MASLD patients. The MASLD patients were older, fatter, and more likely to present hypertension in blood pressure. Through blood biochemical index found that MASLD patients of HDL—C significantly reduced. At the same time, it is worth noting that MASLD patients of FBG, 2 HPG, and HBA1 C were significantly increased, which indicates how MASLD patients blood glucose abnormalities. Meanwhile, ALT, GGT, TBIL, and IBIL were significantly increased only in DH syndrome of MASLD patients. However, TG was significantly increased only in the QD syndrome. In the two syndrome types of DH and QD, most of the differences were not found, only in ALT, the DH group was significantly higher than the QD group (Table 2).

**Table 2:** Characteristics of the discovery cohort

Characteristic		Control (N = 34)	MASLD		*P* (interclass)
			DH (N = 36)	QD (N = 30)	
Age (years)		41.79 ± 16.09	54.19 ± 10.03**	56.40 ± 9.57**	< 0.001
Gender, male (%)		14(41.2)	23(63.9)	15(50.0)	0.159
BMI (kg/m^2^)		22.57 ± 2.90	27.01 ± 3.87**	26.10 ± 3.32**	< 0.001
WHR		0.89 ± 0.05	0.94 ± 0.07*	0.95 ± 0.07*	0.001
BP (mmHg)	SBP	120.65 ± 9.80	133.03 ± 14.90**	130.20 ± 14.36**	< 0.001
	DBP	74.74 ± 8.21	83.97 ± 9.43**	81.83 ± 10.35**	< 0.001
ALT (U/L)		23.53 ± 17.03	35.97 ± 26.23*	25.73 ± 14.39#	0.027
AST (U/L)		23.32 ± 10.42	27.06 ± 18.03	22.04 ± 8.20	0.272
GGT (U/L)		26.91 ± 21.85	44.64 ± 45.01*	30.87 ± 18.70	0.053
HDL-C (mmol/L)		1.27 ± 0.22	1.13 ± 0.34*	1.11 ± 0.22*	0.047
LDL-C (mmol/L)		2.85 ± 0.69	3.06 ± 0.91	3.20 ± 0.93	0.253
TBIL (μmol/L)		12.65 ± 3.14	15.84 ± 5.95*	15.22 ± 5.94	0.029
DBIL (μmol/L)		2.28 ± 0.63	2.61 ± 1.36	3.50 ± 4.98	0.220
IBIL (μmol/L)		10.37 ± 2.65	13.06 ± 5.16*	11.30 ± 6.03	0.063
TG (mmol/L)		1.60 ± 1.79	2.32 ± 1.53	2.54 ± 1.79*	0.067
FBG (mmo/L)		5.07 ± 0.48	8.69 ± 4.11**	8.95 ± 3.68**	< 0.001
2 hPG (mmo/L)		5.07 ± 0.48	12.69 ± 5.16 **	12.71 ± 4.70**	< 0.001
HbA1 C (%)		5.22 ± 0.30	8.83 ± 1.85 **	8.86 ± 2.07**	< 0.001

Compared with the Control group, *P < 0.05

**P < 0.01

#Compared with DH group

*P* < 0.05.

### Changes in tongue image characteristics of dampness-heat syndrome and Qi-deficiency syndrome

After extracting the features of tongue images, the computer tongue image parameters of the two groups of people were obtained. Among the tongue coating thickness indicators, a significant increase in preAll could be observed in the DH group. Comparing the parameters of tongue color and fur color between the two groups, it was observed that the tongue color was darker in the DH group. The specific values are shown in Table 3.

**Table 3:** Tongue image features of participants

	DH (N = 36)	QD (N = 30)	*P*
perAll	0.408 ± 0.144	0.338 ± 0.083**	0.000
TB-R	134.278 ± 12.779	163.800 ± 8.335*	0.032
TB-G	79.417 ± 11.475	97.800 ± 11.090	0.882
TB-B	82.778 ± 11.752	99.833 ± 10.376	0.518
TC-R	123.111 ± 15.131	151.633 ± 13.505	0.788
TC-G	84.389 ± 15.170	103.833 ± 14.879	0.799
TC-B	86.667 ± 14.961	104.333 ± 15.262	0.697
TB-L	40.160 ± 4.669	48.875 ± 3.800	0.272
TB-a	23.471 ± 1.917	26.990 ± 2.727	0.060
TB-b	7.956 ± 1.623	10.398 ± 1.749	0.619
TC-L	40.029 ± 6.064	48.865 ± 5.678	0.960
TC-a	16.506 ± 1.921	19.324 ± 1.949	0.956
TC-b	5.208 ± 1.756	7.541 ± 2.175	0.082

Tongue coating index: perALL; The color index comes from the RGB, Lab, in which R (red value), G (green value), B (blue value), L (lightness), a (red-green axis), b (Yellow-blue axis)

*Compared with DH group, P < 0.05

**Compared with DH group, P < 0.01

**Fig. 4:**
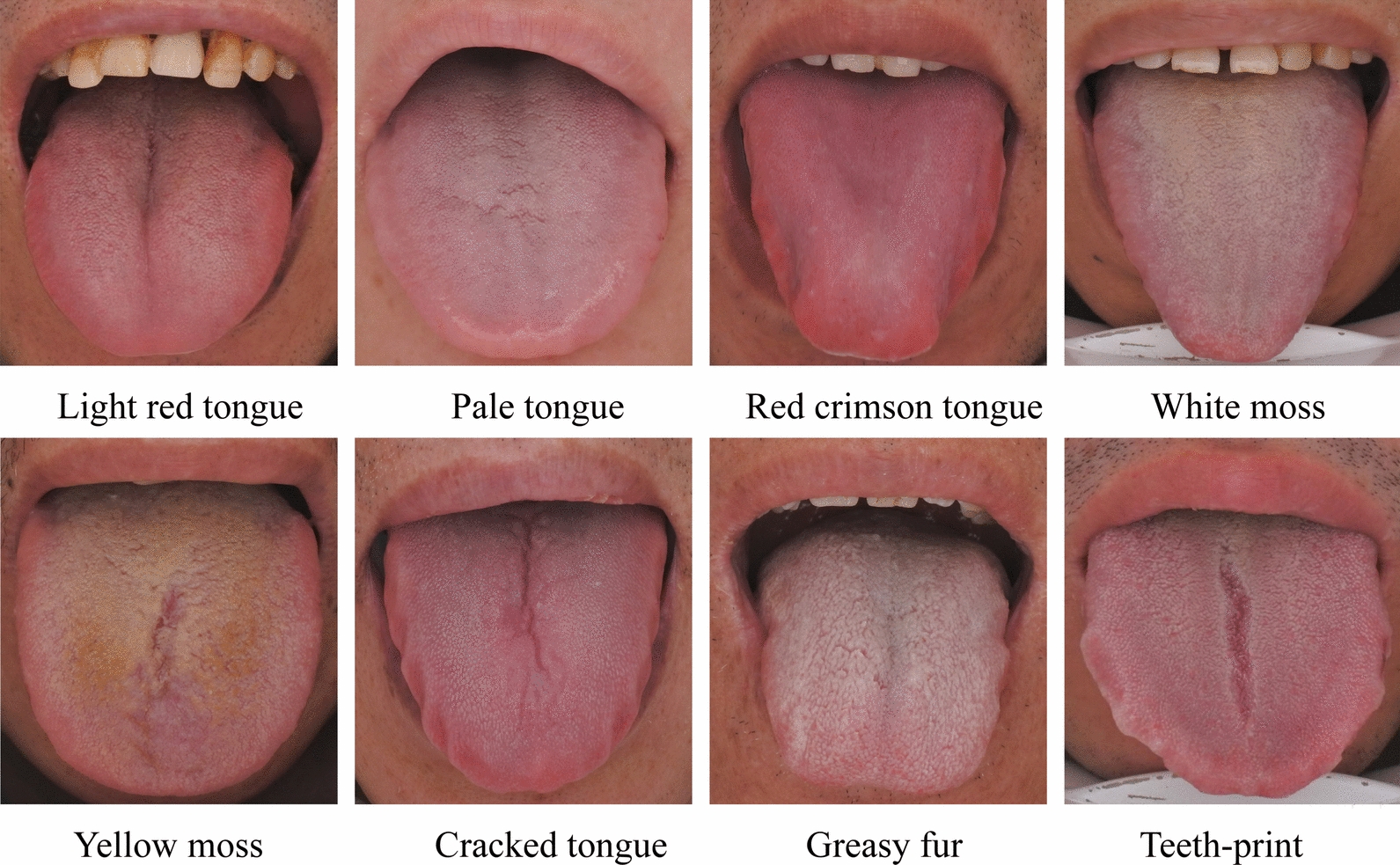
Specific tongue image features

Based on the identification of tongue features by experts and the classification of tongue indicators, the tongue images were classified according to tongue color (Light red, Light white, and Purple-red), moss color (White and Yellow), and tongue texture features (Crack, Teeth-print, Spot, Petechial, Thick fur, Greasy fur, Peeling fur, and Curdy fur). The characteristics of tongue appearance in the DH group and QD group were compared by chi-square test(Table 4). The results showed that the tongue color of DH syndrome patients was red crimson, and the proportion of greasy fur was also significantly increased compared with QD syndrome patients. At the same time, 20% of the patients with QD syndrome showed pale tongue, while no one in the DH group showed such a manifestation, the number of white moss was 27, accounting for 90% of the total, and the proportion of cracked tongue was also significantly higher than that of DH group. The specific image features are shown in Fig. 4.

**Table 4:** Comparison of tongue features between Dampness-Heat syndrome and Qi-Deficiency syndrome

Feature, N = positive (%)		DH (N = 36)	QD (N = 30)	χ2	*P*
Tongue color	Light red	19 (52.8)	19 (63.3)	0.746	0.388
	Pale	0 (0.0)	6 (20.0)	7.92	0.005**
	Red crimson	17 (47.2)	5 (16.7)	6.875	0.009**
Moss color	White	21 (58.3)	27 (90.0)	8.273	0.004**
	Yellow	15 (41.7)	3 (10.0)		
Morphological & textural	Crack	11 (30.6)	19 (63.3)	7.091	0.008**
	Teeth-print	15 (41.7)	14 (46.7)	0.166	0.684
	Spot	2 (5.6)	1 (3.3)	0.186	0.666
	Petechial	7 (19.4)	3 (10.0)	1.135	0.287
	Greasy fur	23 (63.9)	6 (20.0)	12.796	0.000**
	Peeling fur	0 (0.0)	1 (3.3)	1.595	0.207
	Curdy fur	0 (0.0)	3 (10.0)	1.819	0.177

**Compared with DH group, P < 0.01

### Oral microbial profile varied between the dampness-heat syndrome and Qi-deficiency syndrome patient

**Fig. 5:**
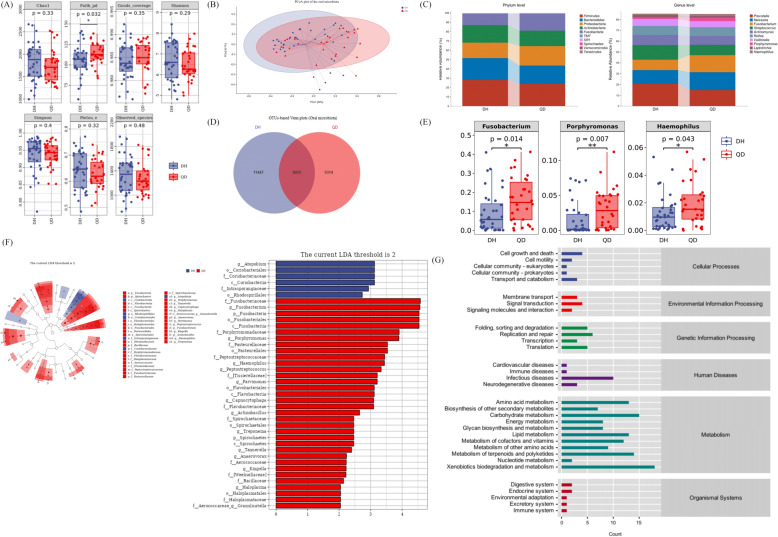
Differences in the relative abundances of oral microbiota between the Dampness-Heat syndrome and Qi-Deficiency syndrome groups. (**A**) The α diversity of oral microbiota; (**B**) PCoA of the oral microbiota; (**C**) Venn diagrams showing the unique and shared species between two groups; (**D**) Stacked bar plots showing the relative abundances of oral microbiota at the phylum level in participants; (**E**) Stacked bar plots showing the relative abundances of oral microbiota at the genus level in participants; (**F**) At the genus level, the flora with statistically significant differences between the two groups (*P < 0.05, **P < 0.01); (**G**) LEfSe analysis of oral signature microbiota in two groups

According to Alpha diversity analysis, Faith's phylogenetic diversity index (Faith_pd) of QD groups in the oral microbiota was significantly higher in the DH group (P = 0.032 < 0.05). However, there were no significant differences in other indices of reaction abundance and evenness index between the two groups(Fig. 5A). Furthermore, the unweighted and weighted Unifrac distances, which are the quantitative measures of the Beta diversity, were analyzed using a PCoA plot which revealed that there were some species differences between DH and QD syndrome groups (Fig. 5B).

Stacked bar charts indicate that, at the phylum level, the most abundant oral microbiota in both the DH syndrome and QD syndrome groups were *Firmicutes* (0.28 and 0.24, respectively). In comparison to the DH group, *Bacteroidetes* exhibited a slight decrease in the QD group (from 0.23 to 0.20), whereas *Proteobacteria* showed an increase in the QD group (from 0.17 to 0.21); At the genus level, *Prevotella* was the most abundant bacterium in the DH group (0.20), and *Neisseria* was the most abundant bacterium in the QD group (0.16) (Fig. 5C). Venn diagram showed that the DH group and QD group had 8055 common microorganisms, accounting for 41.30% and 46.38% of the total oral microorganisms in the two groups, respectively (Fig. 5D). These results showed that the relative abundances of *Fusobacterium, Porphyromonas,* and *Haemophilus* were significantly increased in the QD group(*P* < 0.05 and *P* < 0.01, respectively) (Fig. 5E). Furthermore, the LEfSe-derived cladogram and LDA scores showed that at the genus level, *Atopohium* was abundant in the DH group, whereas *Fusobacterium* and *Porphyromonas* were enriched in the QD group (Fig. 5F).

The aforementioned analyses primarily focused on the diversity and species composition of the microbiota. In the context of microbial ecology research, it is also essential to investigate the functional potential of the microbiota. Consequently, we performed a statistical analysis of metabolic pathways. The results indicated that metabolic-level functions were significantly enriched in the metabolic pathways associated with MASLD DH syndrome and QD syndrome. These encompass xenobiotics biodegradation and metabolism, carbohydrate metabolism, and lipid metabolism (Fig. 5G).

### Association between oral microbiota and tongue image parameters

**Fig. 6:**
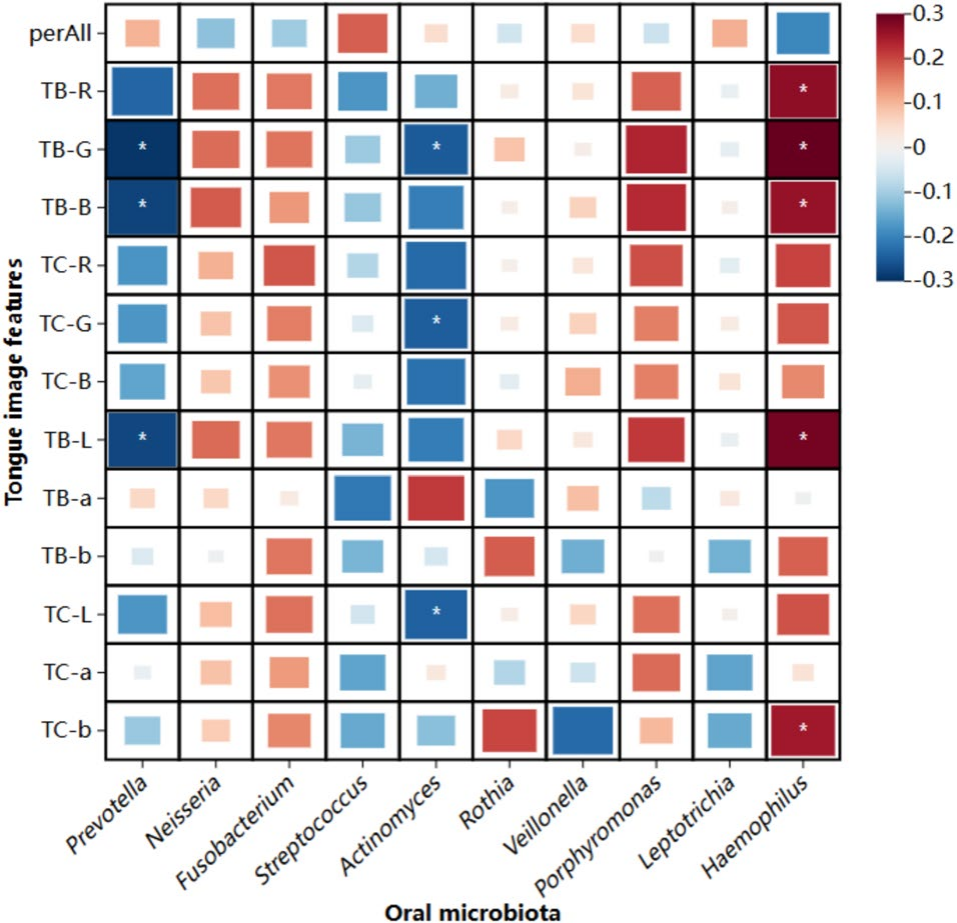
Heat map of the correlation between oral microbiota and tongue image parameters

We proved by Pearson correlation analysis that there is a strong relationship between oral microbiota and changes in tongue image. Among them, *Prevotella*, the bacterium with the highest relative abundance in the oral microbiome, showed a negative correlation with TB-G, TB-B, and TB-L; *Actinomyces* showed a negative correlation with TB-G, TC-G, and TC-L; it is worth noting that *Haemophilus* was positively correlated with TB-R, TB-G, TB-B, TB-L, and TC-b. The heatmap showing the Pearson correlation coefficients between the tongue image metrics and the relative abundance of the top 10 species at the genus level in the oral microbiome is shown in Fig. 6.

### To construct a syndrome classification model of dampness-heat and Qi-deficiency by machine learning technology

**Fig. 7:**
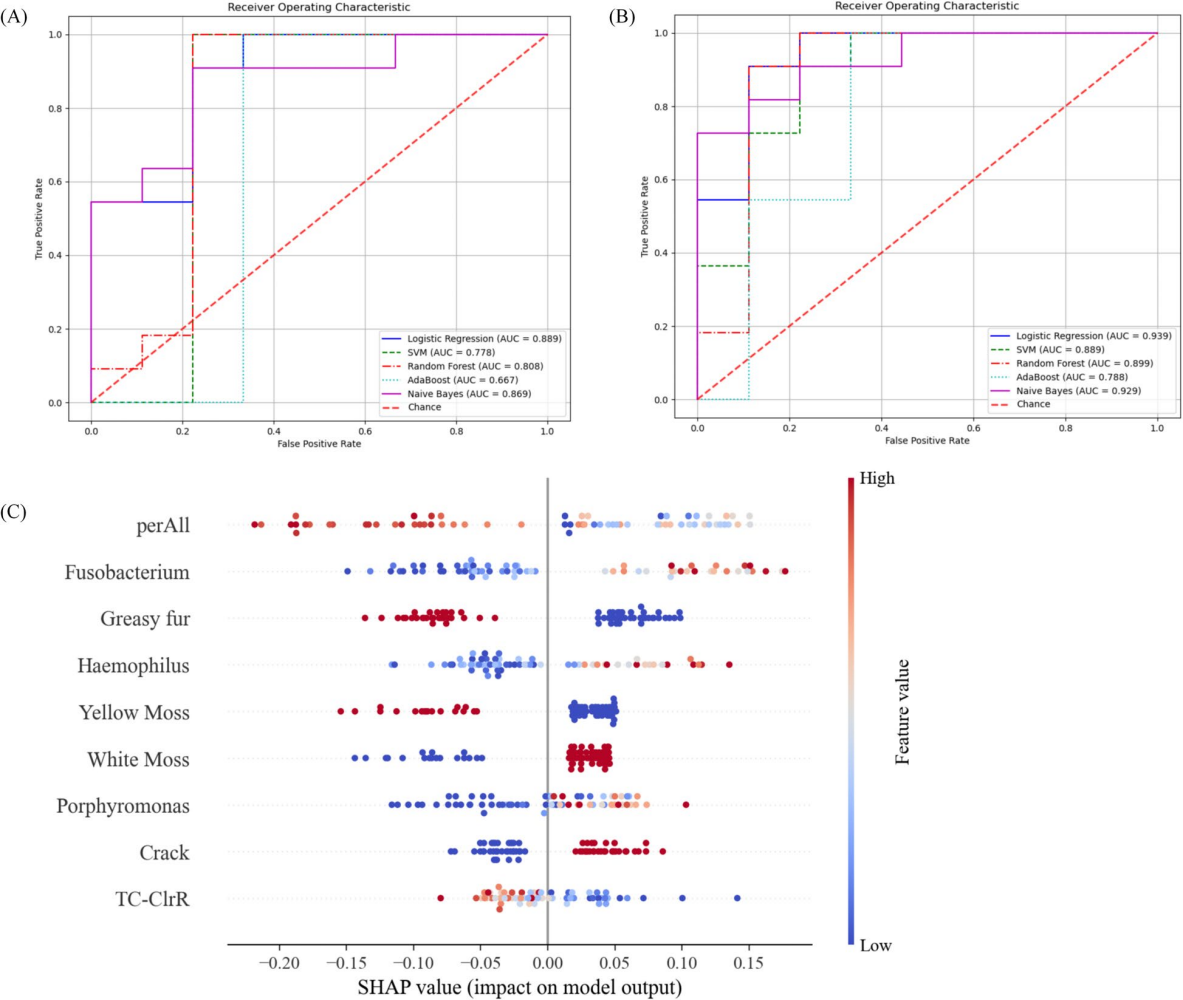
To construct a syndrome classification model of Dampness-Heat syndrome and Qi-Deficiency syndrome groups. (**A**) Tongue image features were used to construct a syndrome classification model of the Receiver Operating Characteristic curve; (**B**)Tongue features combined with oral flora were used to construct a syndrome classification model of the Receiver Operating Characteristic curve; (**C**) SHAP Summary Plot

Based on the prior research, we identified substantial differences in tongue appearance between the two syndromes, as well as distinct landmark bacterial genera. Consequently, we aimed to develop a MASLD syndrome classification model with tongue features serving as the core component. Compared with tongue features alone, the combination of tongue features and oral flora is more effective in the classification of the two syndromes. In logistic regression modeling, the receiver operating characteristic (ROC) curves showed that tongue characteristics could distinguish DH and QD syndrome types in MASLD patients, the AUC value was 0.889, and the Accuracy was 0.750 (Fig. 7A,). After adding *Fusobacterium*, *Porphyromonas*, and *Haemophilus*, the Accuracy of logistic regression was increased to 0.850, and the AUC value was 0.939 (Fig. 7B, Table 5). The accuracy of other models increased, among which the model with the lowest Accuracy was AdaBoost (0.650) and the AUC value was 0.788 (Fig. 7B, Table 5). According to the feature importance evaluation of random forest, it was found that perALL of the tongue image parameters contributed the most to the prediction results (41.79%), followed by *Fusobacterium*(15.17%) and *Porphyromonas*(12.42%) in the oral flora (Fig. 7C).

**Table 5:** Comparison of diagnostic efficiency of five different machine learning methods

Variables	Methods	AUC	Accuracy	Precision	Recall	F1-score
Tongue	Logistic regression	0.889	0.750	0.800	0.727	0.762
	SVM	0.778	0.750	0.800	0.727	0.762
	Random forest	0.813	0.700	0.778	0.636	0.700
	AdaBoost	0.667	0.750	0.750	0.818	0.783
	Naive bayes	0.869	0.800	0.818	0.818	0.818
Tongue & oral flora	Logistic regression	0.939	0.850	0.833	0.909	0.870
	SVM	0.889	0.800	0.818	0.818	0.818
	Random forest	0.899	0.900	0.909	0.909	0.909
	AdaBoost	0.788	0.650	0.700	0.636	0.667
	Naive bayes	0.929	0.800	0.818	0.818	0.818

## Discussion

Syndrome differentiation and treatment is the basic principle of TCM research, and the syndrome is the general term for the occurrence and development of diseases, which is the premise and basis of TCM diagnosis and treatment [22]. Disease-location syndrome elements and disease-characteristic syndrome elements are the important components of TCM syndrome elements, among which disease-characteristic syndrome elements include wind, cold, summer, damp, dryness, fire, heat, etc. As a metabolic disease, MASLD is believed to be closely related to striking dampness [22, 23]. Our results also showed that among the 84 MFALD patients collected, the most patients were Dampness-Heat syndrome, with 36 patients, followed by Qi-Deficiency syndrome, with 30 patients.

In clinical practice, TCM syndrome type often changes according to the aggravation of the disease [24]. In the statistical analysis of biochemical indicators of patients, ALT in the DH group was significantly higher than that in the QD group (*P* < 0.05), while the index reflecting liver function level was only significantly increased in DH syndrome patients. So we probably infer QD type certificate is an early stage in MASLD, and liver function status has no obvious damage. This observation is also similar to the results of other studies [4, 25].

Tongue diagnosis is one of the most common and basic diagnostic methods followed by TCM diagnosis [26]. The characteristics of tongue image include tongue shape, tongue color, tongue texture, tongue back, coating color, thickness of the coating, etc. [27]. Normal people usually have tongue light red and a thin white coating [28]. In previous experiments, we have demonstrated that tongue features change significantly in MASLD [12]. Meanwhile, the tongue image changes with the occurrence of positive or deficiency syndrome. In this study, preAll in the DH group was significantly higher than that in the QD group, indicating that the tongue coating in the DH group was thicker and greasy, and the tongue coating color was more yellow than that in the QD group. At the same time, the tongue color and fur color of the QD group were lighter than that of the DH group. In TCM, the pale tongue is related to deficiency of heart and spleen, deficiency of heart, gallbladder, and qi, and deficiency of heart and timidity. In the experiments of other scholars, it can also be found that the tongue color of patients with qi and blood deficiency syndrome is pale [29]. At the same time, patients with excessive damp-heat in the body are often accompanied by yellow and greasy tongue coating, and similar results can be seen in intestinal damp-heat syndrome [30, 31].

Accumulating evidence indicates that oral microbiomes influence the human microbial community and human health and can be a diagnostic too [32, 33]. In this study, some differences in species composition and abundance were found between the two syndromes of MASLD. These results confirm that even in the same disease, there will be different microbial differences under different syndrome types. The value of syndrome classification is proved from the microscopic point of view.

In terms of species composition, *Prevotella* was the most abundant bacterium in the DH group, and *Neisseria* was the most abundant bacterium in the QD group. In the study by Sunmin Park et al. [34], enterotypes dominated by *Prevotella* species were found to be at high risk for MASLD in Asian populations. *Prevotella* can produce higher levels of lipopolysaccharide (LPS), which triggers inflammation and promotes the development of MASLD [35, 36]. *Neisseria* are generally Gram-negative organisms and are mainly non-pathogenic in the oral cavity [37]. The genus *Neisseria* was also demonstrated to be one of the major “mouth shapes” in MASLD patients in the experiment of Xiaodong Li et al. [38]. *Fusobacterium* and *Porphyromonas* may be the key strains to distinguish the DH syndrome type from the QH syndrome type. *Fusobacterium nucleatum*, an anaerobic resident bacterium in the mouth, is thought to contribute to ulcerative colitis [39]. Previous studies have shown that *Fusobacterium* is closely related to the production of yellow fur, which is similar to the results of this experiment [38]. *Porphyromonas* is a major pathogen of chronic periodontitis [40], and some experiments have shown that *Porphyromonas* induces IL-17 and IL-10-mediated inflammatory responses [41]. These results further suggest that changes in syndrome type can alter the compositional changes of microbial communities at different taxonomic levels. By exploring the metabolic pathway between the two syndromes, we can also find that most of the oral flora causing the DH syndrome and QD syndrome of MASLD are enriched in Metabolism, which also confirms that MASLD as a metabolic disease, has metabolic changes in oral flora.

By exploring the characteristic parameters of oral microbiota and tongue image, changes in oral microbiota were strongly correlated with changes in tongue image. We found that *Prevotella* showed a negative correlation with TB-G, TB-B, and TB-L. Meanwhile, *Haemophilus* was positively correlated with TB-R, TB-G, TB-B, TB-L and TC-b. R*G*B is an important indicator of tongue color. However, in the characteristic parameters of tongue image, the R*G*B value of the QD group was higher than that of the DH group, which may be due to the thick tongue coating of the DH group, which covered the original tongue color. The TB-L value is an important marker of tongue lightness. The significant increase in the abundance of *Haemophilus* in the QD group means that the tongue color of the QD group is brighter than that of the DH group, which means that the patients in the DH group have a darker tongue color. *Haemophilus* is a widespread genus of bacteria, abundant in most oral sites, and one of the most transcriptionally active species in the human oral cavity [42, 43]. In an experiment on the early diagnosis of cancer by tongue examination, *Haemophilus* was also found to be more abundant in healthy subjects with reddish tongues than in cancer subjects with reddish tongues [44].

In this experiment, a syndrome classification method based on tongue image features was established, and good accuracy was achieved (AUC = 0.889), which confirmed that tongue diagnosis played a major role in syndrome classification. Machine learning models based on tongue features and microbiota also play a good role in predicting the risk of pre-diabetes and type 2 diabetes [45]. In the classification of DH syndrome and QD syndrome, especially the thickness of tongue coating is the most important difference in tongue image. Similar results were obtained in the identification of TCM syndromic characteristics of patients with erosive gastritis [33]. At the same time, this experiment confirmed that the machine learning model of tongue image features combined with oral flora had better classification ability (AUC = 0.939), and could effectively classify MASLD patients into DH syndrome type and QD syndrome type. Among them, the iconic bacteria *Fusobacterium*, *Porphyromonas,* and *Haemophilus* all played an important role. These findings further demonstrate that oral microbes can be used as biomarkers for the diagnosis of diseases and syndromes [15, 46]. Because tongue features are subject to too many human factors, and our previous research found that tongue body characteristics can change with circadian rhythm [47], which makes it difficult to maintain accuracy and objectivity [48]. Compared with biochemical indicators and tongue images, oral flora has advantages in the simplicity of collection and objectivity of experiment [49]. The key next step is to generalize and transform these critical microbial biomarkers into available tools for clinical practice.

A limitation of this study was the limited sample size. Although the inclusion criteria were rigorous, future investigations with a larger sample size were needed to control for potential confounding factors. Only the population characteristics of two main syndromes were explored. In the future, the sample size will be expanded to explore the syndrome characteristics of Phlegm-Turbid syndrome and Phlegm-Stasis syndrome. The goal of this study is to establish a MASLD syndrome classification model with tongue characteristics as the core, and the content of intestinal flora will be added in the future, hoping to construct a disease and syndrome diagnosis model with microbial characteristics as the core. Moreover, further studies are needed to investigate the underlying mechanism of different tongue features in future metabolomics experiments.

In conclusion, we comprehensively described the tongue image change characteristics and oral microbiome changes between the “Dampness-Heat” and “Qi-Deficiency” syndrome types in MASLD patients, explored the correlation between tongue image characteristics and oral flora, and evaluated the potential value of oral microbiome as an auxiliary diagnostic tool for syndrome classification. This study preliminarily confirmed that tongue image features are related to microbial metabolism, which can provide the theoretical basis for the objective analysis of tongue diagnosis for the classification of syndrome types.

## Data Availability

The research data generated from this study are included within the article and additional files.

## Abbreviations

2hPG: 2-hour postprandial blood glucose
ALT: Alanine Aminotransferase
AST: Aspartate Aminotransferase
AUC: area under the curve
BMI: Body Mass Index
DBIL: Direct bilirubin
DH: Dampness-Heat
FBG: Fasting blood glucose
GGT: Gamma-GlutamylTransferase
HbA1C: Glycosylated hemoglobin
HDL-C: High-density lipoprotein cholesterol
IBIL: Indirect bilirubin
LDL: Low-density lipoprotein cholesterol
LEfSe: Linear discriminant analysis effect size
LPS: lipopolysaccharide
MASLD: metabolic dysfunction-associated steatotic liver disease
PCoA: principal coordinate analysis
QD: Qi-Deficiency
ROC: receiver operating characteristic
SVM: support vector machine
TBIL: Total bilirubin
TCM: Traditional Chinese medicine
TG: triglyceride
UACANet: Uncertainty Augmented Context Attention Network
WHR: Waist-hip ratio
